# A Case of Acrodermatitis Dysmetabolica in a Child Affected by Citrullinemia Type I: When Early Diagnosis and Timely Treatment Are Not Enough

**DOI:** 10.3390/children10091491

**Published:** 2023-08-31

**Authors:** Laura Bruni, Alessandra Cassio, Valeria Di Natale, Federico Baronio, Rita Ortolano, Andrea Pession, Bianca Maria Piraccini, Iria Neri

**Affiliations:** 1 Specialty School of Pediatrics-Alma Mater Studiorum, University of Bologna, 40126 Bologna, Italy; laura.bruni4@studio.unibo.it; 2 Department of Medical & Surgical Sciences, University of Bologna, 40138 Bologna, Italy; alessandra.cassio@unibo.it; 3 Pediatric Unit, IRCCS Azienda Ospedaliero-Universitaria di Bologna, 40138 Bologna, Italy; valeria.dinatale@aosp.bo.it (V.D.N.); federico.baronio@aosp.bo.it (F.B.); rita.ortolano@aosp.bo.it (R.O.); andrea.pession@unibo.it (A.P.); 4 Unit of Dermatology, IRCCS di Policlinico S. Orsola, 40126 Bologna, Italy; biancamaria.piraccini@unibo.it; 5 Division of Dermatology, Department of Experimental, Diagnostic and Specialty Medicine, University of Bologna, 40138 Bologna, Italy

**Keywords:** urea cycle disorders, acrodermatitis dysmetabolica, compliance

## Abstract

An infant with a prenatal diagnosis of citrullinemia, who started standard treatment at birth (L-arginine; sodium benzoate and a personalized diet characterized by a low protein intake and supplementation of essential nutrients and amino acids), presented at 4 months of age with extended, progressive, and severe skin lesions consistent with acrodermatitis dysmetabolica. Guidelines for the diagnosis and management of urea cycle disorders underline that a low-protein diet places patients at risk of essential fatty acids, trace elements, and vitamin deficiency. At hospital admission, our patient had normal levels of zinc and alkaline phosphatases. The plasmatic amino acid profile revealed a severe and generalized deficiency. In particular, the serum levels of arginine, valine, and isoleucine were very low and the dermatitis did not improve until the blood levels of these amino acids increased. In our patient, skin lesions happened despite an early diagnosis of citrullinemia and timely treatment due to compliance issues as a consequence of linguistic barriers.

## 1. Introduction

Urea cycle disorders (UCDs) are inborn errors of metabolism due to defects in any of the enzymes or transporter molecules involved in the hepatic removal of ammonia from the bloodstream. With a 1:35.000 estimated incidence, UCDs are some of the most common inborn metabolic diseases.

Citrullinemia type I (CTLN1) is a rare autosomal recessive UCD caused by a deficiency or absence of the enzyme argininosuccinate synthetase (ASS). It can present as a neonatal acute (classic) form or a milder, late-onset form. Women presenting the onset of symptoms at pregnancy or post-partum and cases without symptoms or hyperammonemia have also been described. CTLN1’s main clinical hallmark is hyperammonemic crisis, which is mostly triggered by the change from intrauterine to neonatal life and catabolic events, protein overload, or the intake of certain drugs.

Infants affected by the acute neonatal form seem to be normal at birth, but when the disease is not recognized immediately and left without timely treatment, they very soon develop hyperammonemia, which, in turn, causes encephalopathy, spasticity, seizures, and even death.

Timely diagnosis and treatment are essential to preventing cognitive impairment, which correlates with the duration and severity of the hyperammonemia. However, even when promptly and adequately treated, children with the severe form may show neurological deficits over time and liver failure can be a long-term complication.

The only curative treatment currently available is liver transplantation (to be performed possibly in the first year of life to prevent neurocognitive impairment).

The daily treatment for children who cannot be transplanted or are waiting to undergo a liver transplantation aims at maintaining stable metabolic control, preventing chronic complications, and achieving normal development and growth. It includes a combination of drugs to increase waste nitrogen excretion, a low-protein diet, the supplementation of arginine and/or citrulline, the supplementation of essential nutrients such as vitamins, minerals, and essential amino acids, and an emergency regimen for the treatment of intercurrent illnesses.

As underlined in the recent suggested guidelines for the diagnosis and management of UCDs, the low-protein diet places UCD patients at risk of essential fatty acid, trace elements, and vitamin deficiency [1].

Since 2016 (Law 167/2016), in Italy, UCDs, including CTLN1, are included in the panel of mandatory newborn screening (NBS). Thanks to this important public health program, it is now possible to identify a child affected by CTLN1 at birth, take care of them, and immediately start treatment and adequate follow up, hopefully changing the course of the disease and improving the quality of life of the child and their family.

## 2. Clinical Case

We describe the case of an infant with a prenatal diagnosis of CTLN1 who presented at 4 months of age with extended, progressive, and severe skin lesions.

Our patient is a full-term female, born to consanguineous parents (first cousins) originally from Pakistan. She received a prenatal diagnosis of CTLN1 for positive familiarity (older brother previously diagnosed through NBS as being affected by the same disease). Since birth, she was on standard treatment with L-arginine, sodium benzoate, and a personalized diet characterized by a low protein intake and the supplementation of essential nutrients (vitamins and minerals) and essential amino acids. In the first month of life, she was supported with physical feeding therapy due to feeding difficulties and an initial reduced food tolerance.

During her regular follow ups at our Metabolic Centre, she always had normal or slightly elevated ammonia levels and never experienced a hyperammonemic crisis. When asked, the parents never reported feeding difficulties.

At 4 months of age, she was admitted to our Metabolic Centre for the evaluation of a skin rash, the onset of which was approximately 1 week before, following 2 days of gastroenteritis. The lesions were initially noted on the diaper area and progressively extended.

**Figure 1:**
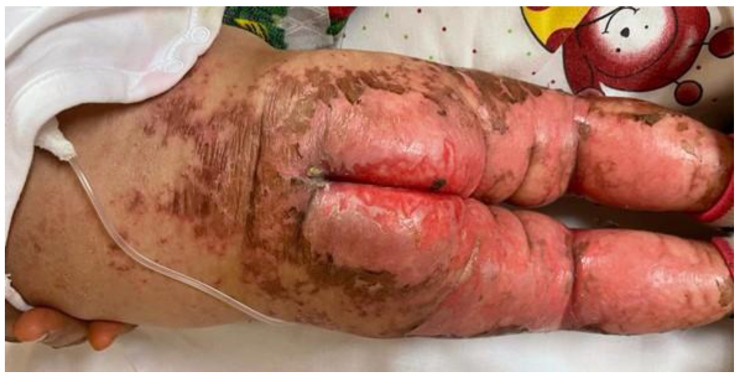
Erythematous desquamative patches with large, combustiform, scaly lesions at hospital admission: back, diaper area, and legs.

**Figure 2:**
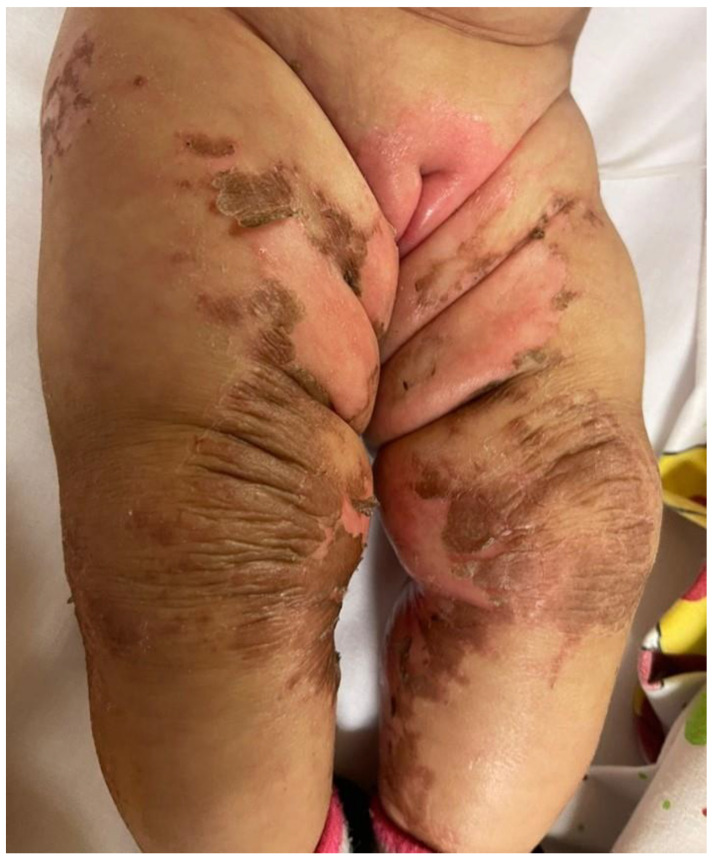
Erythematous desquamative patches with large, combustiform, scaly lesions at hospital admission: diaper area and legs.

**Figure 3:**
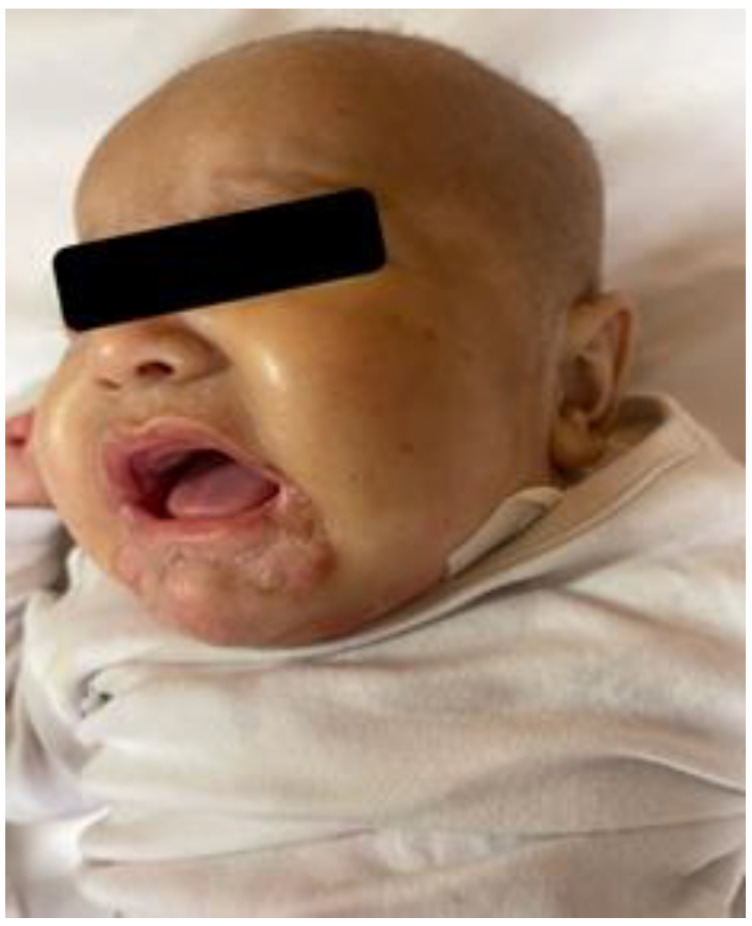
Erythematous desquamative patches with large, combustiform, scaly lesions at hospital admission: perioral region.

She was in poor general conditions, afebrile but dehydrated and suffering. The physical examination showed erythematous desquamative patches with large, combustiform, and scaly lesions involving the diaper area, trunk, perioral region, neck, and extremities. Ulcerations in the perianal area were also present, as shown in Figure 1, Figure 2 and Figure 3. Her body weight was 4.050 Kg (far below the 3rd WHO centile), the same as 1 month before.

At hospital admission, her theoretical prescribed diet consisted of 7 meals of 90 mL of formula, providing a total liquid intake of 158 mL/kg/day, energy intake of 136 kcal/kg/day, and protein intake of 1.6 g/kg/day (natural proteins 0.9 g/kg and synthetic proteins 0.7 g/kg), fulfilling the vitamin and mineral requirements for her age.

However, investigating the medical history more in depth, we discovered that the infant still had feeding difficulties and rarely completed her meals. The parents had never reported these issues before as, due to linguistic difficulties, they had not fully understood our prescription and the implication of not following it correctly. 

The blood exams showed mild anaemia (Hb 9.1 g/dL, n.v. 9.7–13.4; HTC 26%, n.v. 29–40.5; MCV 76 fL, n.v. 78–103); a normal white cell count; normal markers of inflammation; low urea (<5 mg/dL; n.v. 8–26), creatinine (0.12 mg/dL; n.v. 0.5–1.2), total proteins (3.5 g/dL; n.v. 5.7–8), and albumin (20.3 g/L; n.v. 35–50); normal liver function; no acidosis; and an elevated ammonia level (124 μmol/L; n.v. < 53). An evaluation of plasmatic amino acid level was performed.

Due to a high level of procalcitonin the day after admission (91 pg/mL), associated with a skin swab positive for *P. aeruginosa*, *C. albicans*, *S. aureus*, and *E. coli*, even in the absence of impetiginisation, an intravenous antibiotic (piperacillin tazobactam, clindamycin) and antimycotic (fluconazole) therapy was started. Blood cultures always remained negative.

A dermatological consultation hypothesized an acrodermatitis enteropathica-like eruption in her metabolic disorder. The blood tests showed a slightly reduced level of zinc (79 μg/dL; n.v. 90–120); normal alkaline phosphatases (214 U/L; n.v. 124–341) and vitamin B12 (1042 pg/mL; n.v. 145–914); and low levels of copper (31 μg/dL; n.v. 80–155) and iron (29 μg/dL; n.v. 60–180). The plasmatic amino acid profile revealed a severe and generalized deficiency, as shown in Table 1.

The trunk and limbs lesions were treated with the topical application of a basic cream; the perianal erosions were treated at first with a gauze with iron nitrate and then with fusidic acid. To soothe the pain caused by the skin lesions, analgesic therapy with morphine was settled until the resolution of the ulceration.

From a metabolic point of view, at admission, the infant was rehydrated parenterally.

During the first days of hospitalization, the ammonia levels showed an increasing trend that requested intravenous detoxifying therapy and a disruption of oral protein intake, that was later reintroduced gradually. Considering the low proteins and albumin blood levels, the infant received an intravenous infusion of albumin until normalization. She always showed difficulties in finishing her meals.

**Figure 4:**
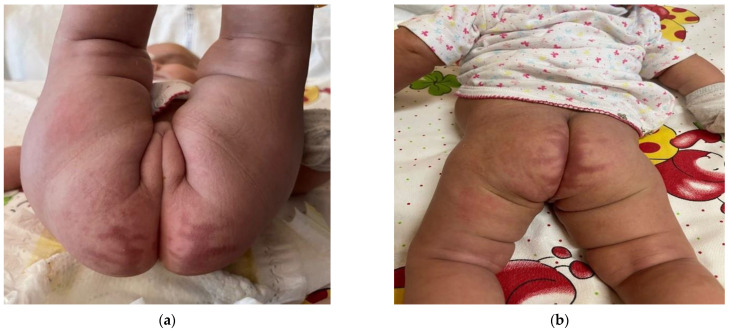
Resolution of skin lesion at hospital discharge: diaper area (**a**) and legs (**b**).

To improve her food tolerance, at 4 and a half months of age, she started weaning with solid foods. Sodium benzoate, which could cause inappetence as a possible side effect, was replaced with glycerol phenylbutyrate with benefit. The infant was discharged at 5 months of age in good clinical conditions, with a complete resolution of the dermatitis, as shown Figure 4, a good food tolerance, and a weight gain of 750 g in 30 days. Instructions for the future were given and a follow up appointment was booked. The amino acid blood levels at hospital discharge are shown in Table 1.

## 3. Discussion

Acrodermatitis enteropathica-like conditions are associated with an acquired zinc deficiency (mainly due to inadequate zinc intake, intestinal malabsorption, increased urinary zinc excretion, and increased requirements) or a disorder in amino acid or fatty acid metabolism. The association between acrodermatitis enteropathica-like eruptions and metabolic diseases (Propionic acidaemia, Methylmalonic acidaemia, Glutaric aciduria type 1, Multiple carboxylase deficiency, Maple syrup urine disease, Phenylketonuria, Ornithine transcarbamylase deficiency, and Carbamoyl phosphate synthetase deficiency) is also described in the literature.

There are only a few cases, published many years ago, describing severe skin lesions in patients affected by CTLN1 [2,3]. At that time, NBS for citrullinemia was still not available, and it was more difficult to diagnose this disease early. For this reason, it was more likely that a patient with an inborn error of metabolism presented with acute and severe disease, including uncommon manifestations such as skin involvement. This was the case of the two children described by Thoene et al. in 1986 and Goldblum et al. in 1977. These children presented, before the diagnosis of citrullinemia, with cutaneous findings similar to those shown by our patient.

In the child described by Thoene, a weeping dermatitis was noted in the third week of life (prior to diagnosis and so before an adequate dietary treatment was established), while the child was being treated with volume exchange transfusions due to hyperammonaemia and coma. Then, once the diagnosis of CTLN1 was made, the child started adequate dietary treatment. However, at 50 days of life, concurrently with the accidental omission of arginine, histidine, and lysine from the ketoacid–amino acid mixture, for four days, the child developed a scaling, weeping, and erythematous dermatitis more severe than the initial eruption. When measured, the level of arginine in the plasma was undetectable. After increasing the dosage of arginine in the mixture, and in parallel with an increased plasma arginine concentration, the clinical signs improved and resolved in 48 h.

Goldblum described the very similar case of a child who developed neurological signs within the first days of life and progressed to coma. Due to hyperammonaemia, he underwent peritoneal dialysis with an improvement in his clinical conditions. Then, he started treatment with sodium benzoate and a low-protein rice formula plus corn syrup and vegetable oil. Starting from day 35 of life, he developed a generalized eruption consisting of generalized exfoliative, scaly, moist, erythematous, and eroded patches and plaques that were most prominent in the perioral region, lower abdomen, diaper region, and buttocks. The plasma arginine level was significantly below normal values. At the same time, a diagnosis of CTLN1 was made on the basis of hyperammonemia and the elevated citrulline level in the plasma. A formula supplying protein plus arginine supplementation, sodium benzoate, and sodium phenylacetate was started, with a very fast improvement and resolution of the cutaneous lesions.

Both authors linked the skin involvement to arginine deficiency due to the underlying metabolic disease and inadequate diet. In fact, arginine is an important constituent of human epidermal keratin [4]. We support this hypothesis: our patient had very low levels of arginine at hospital admission and the dermatitis did not improve until food tolerance was achieved and the blood levels of arginine increased. Moreover, our patient showed low levels of many other essential amino acids, including isoleucine and valine, also considered to be possible causes of keratinocyte growth arrest [5].

More recently, the term acrodermatitis dysmetabolica has been proposed for acrodermatitis enteropathica-like eruptions unrelated to zinc deficiency and described in metabolic disorders, such as methylmalonic acidemia, propionic acidemia, maple syrup urine disease, glutaric aciduria type I, ornithine transcarbamylase deficiency, and citrullinemia. This acrodermatitis seems to be caused by a deficiency of essential amino acids and fatty acids [6,7,8]. It has been suggested that these metabolites are essential for the proliferation and cell differentiation of keratinocytes, and their depletion could explain the necrosis [5]. The current guidelines for the diagnosis and management of UCDs underline that the low-protein diet places patients at risk of essential fatty acids, trace elements, and vitamin deficiency [1]. 

Some other cases have been already described in the literature and our case does not add too much to either the description of the lesions or the hypothesis for the pathogenesis of the disease [2,3,6,7,8]. Nevertheless, we decided to describe this patient as, to our knowledge, it is the first case reported in the literature in which skin lesions happened despite an early (at birth) diagnosis of CTLN1 and timely dietary treatment.

Actually, our patient theoretically already had an indication for specific treatment and a balanced diet, and these severe manifestations could have been prevented. In fact, it can be argued that, with an older sibling affected by the same disease, the family should have already been trained to deal with the specific diet and treatment. However, the first child presented with an acute encephalopathy, the onset of which was before the result of NBS. For this reason, he spent most of his first months of life in hospital, managed (including diet) directly by healthcare professionals.

This case also underlines the difficulty of achieving a good compliance to treatment when there are linguistic barriers. Due to a previous bad experience happening with the older son, the parents refused our proposal to involve a cultural mediator to help them understand the information we were going to give them (they attributed to the cultural mediator the responsibility of having violated the professional confidentiality and shared with their community important and confidential information about the disease of their son, causing them, de facto, marginalization by the community itself). Without the support of a cultural mediator and despite our efforts to explain them that the diet was of huge importance for the treatment of their daughter, they did not fully understand and did not pay too much attention to the small but significant quantities of formula left apart by her at each meal. Furthermore, they underestimated the clinical condition and dermatitis of the infant, and they sought medical attention quite late. When our patient was discharged, social services were contacted to support the family and improve their compliance. A home nursing assistant is currently weighing the baby twice a week and verifying that she is given the correct amounts of food.

## 4. Conclusions

This is a case of a very rare and severe complication of an ultra-rare disease, which happened despite the early diagnosis of citrullinemia and its timely treatment. Poor compliance to the dietary regimen, due to linguistic barriers, seemed to be the major cause (if not the only one) of such a complication. In fact, the dermatitis improved when the amino acid profile normalized as a consequence of the correct and controlled feeding during hospitalization. 

This case underlines the importance to always having a careful approach to the family, particularly when there are linguistic barriers, keeping in mind that some people could have a different perception of the illness and ignore or misunderstand the importance and the meaning of the prescribed therapies.

## Figures and Tables

**Table 1:** Plasmatic amino acid profile at hospital admission and hospital discharge. Values below the reference value are marked with ↓; values above the reference value are marked with ↑.

Amino Acid	Patient Value (μmol/L) Admission to Hospital	Patient Value (μmol/L) after 1 Month	Reference Value 1–6 Months (μmol/L)	Reference Value (μmol/L) from Our Court
Taurine	85	185	29–143	39–111
Asparagine	8	6	<40	<10
Threonine	44 ↓	139	102–176	61–163
Serine	84 ↓	87 ↓	111–165	97–168
Glutamic acid	21 ↓	70		26–78
Glutamine	684 ↑	369 ↑		363–524
Proline	81 ↓	75	135–239	47–256
Glycine	218	162 ↓	182–264	145–348
Alanine	294	291	253–427	182–482
Citrulline	1556 ↑	2068 ↑	20–34	14–39
Valine	52 ↓	117 ↓	186–264	167–283
Cysteine	4 ↓	34	32–50	28–51
Methionine	17 ↓	29	24–38	15–26
Isoleucine	22 ↓	51 ↓	56–90	45–80
Leucine	34 ↓	67 ↓	107–157	77–152
Tyrosine	27 ↓	60	59–101	36–74
Phenylalanine	20 ↓	14 ↓	49–67	43–61
Ornithine	6 ↓	47 ↓	59–99	40–87
Lysine	47 ↓	132 ↓	153–239	112–182
Histidine	52 ↓	44 ↓	69–101	62–95
Arginine	3 ↓	54 ↓	56–104	43–120

## Data Availability

Not applicable.
